# Epitaxial Mixed-Dimensional MoS_2_ Nanofin-Nanoribbon Hybrids and Their Integration into Electronic and Optoelectronic Devices

**DOI:** 10.1021/acsami.5c00308

**Published:** 2025-04-30

**Authors:** Yarden Danieli, Lothar Houben, Katya Rechav, Olga Brontvein, Ifat Kaplan-Ashiri, Iddo Pinkas, Ayelet Vilan, Ernesto Joselevich

**Affiliations:** † Department of Molecular Chemistry and Materials Science, Weizmann Institute of Science, Rehovot 7610001, Israel; ‡ Department of Chemical Research Support, Weizmann Institute of Science, Rehovot 7610001, Israel

**Keywords:** MoS_2_, photodetectors, finFET, mixed-dimension, epitaxy

## Abstract

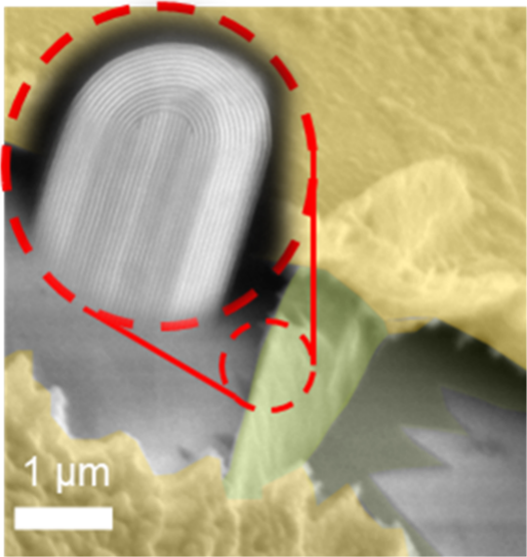

Transition metal dichalcogenides, notably MoS_2_, have garnered substantial attention owing to their excellent optical and electrical properties. While various methods have been employed to grow MoS_2_, resulting in nanostructures with diverse dimensionalities, controlling the lattice orientation and synthesizing aligned nanostructures beyond 2D remain a formidable challenge. In this study, we report the epitaxial growth of aligned MoS_2_ nanofin-nanoribbon hybrids, each consisting of a horizontal nanoribbon with a vertical lamellar structure (“fin”) in its center. Structural analysis reveals epitaxial relations that induce the growth into three isomorphic orientations following the 3-fold symmetry of the *C*-plane sapphire substrate. The nanofin-nanoribbon hybrid was integrated into a fin-channel photodetector with response times on the scale of tens of μs and high photocurrent. Furthermore, the nanofin-nanoribbon hybrids are incorporated into n-type “fin-FET” transistors, showing on–off ratios on the order of ∼10^3^ at room temperature. The performance of these devices is discussed in terms of the efficient fabrication process, devoid of postgrowth steps, and the unique dimensionality of the device, which realizes a high optical path in the fin-shaped channel. This work demonstrates the integration of MoS_2_ into efficient fin-channel electronic and optoelectronic devices, laying the foundation for large-scale integration of TMDs into devices with nonstandard channel configurations.

## 1 Introduction

Since the first determination of their structure in 1923 by Linus Pauling,^1^ transition metal dichalcogenides (TMDs), a noteworthy class of two-dimensional (2D) van der Waals materials, have become a promising materials group for solid-state devices. Their unique physical properties, such as layer-dependent band structure,^2^ tightly bound excitons,^3^ and high current on–off ratio,^4^ were already manifested successfully in various electrical and optoelectronic devices as field-effect transistors (FETs),^4^ photodetectors,^5^ solar cells,^6^ and light-emitting diodes (LEDs).^7^ One frequently explored member of this family is MoS_2_, composed of covalently bound sulfur-sandwiched molybdenum atoms, forming a single layer reaching 6.5 Å thickness.^5^ This monolayer exhibits strong quantum confinement effects, leading to a direct bandgap of 1.8 eV.^8^ Conversely, multilayered MoS_2_ adopts an indirect band gap of 1.2 eV.^8^ The range between these two extrema tunes the MoS_2_ band structure due to the layer number and, thus, the crystal thickness. Although monolayered MoS_2_ is considered a promising candidate in optoelectronics, few to multilayered MoS_2_ can also show a particular optical response that makes this material suitable for optical devices.^9^

Chemical vapor deposition (CVD) has emerged as a promising technique for creating many different structures of MoS_2_, including the abundant form of triangular 2D crystals.^10^ One attractive way to control and tune the material’s physical properties is by controlling its dimensionality. Alongside the ongoing research regarding materials with specific dimensional characteristics, there is a growing understanding that by combining different dimensionalities within the same system, whether through the creation of heterostructure^11^ or by inducing distinct dimensional features within a single nanomaterial,^12^ novel physical properties and functionalities may manifest.^13,14^ These materials are collectively referred to as “mixed-dimensional nanomaterials”.^15^ In this context, different CVD syntheses allowed the creation of mixed-dimensional nanomaterials, holding dimensional features of 2D and 1D structures, such as MoS_2_ nanotubes,^16^ nanoribbons,^17^ nanowires,^18^ or vertically standing nanosheets.^19^ However, in most cases, the synthesis process yields randomly oriented MoS_2_ crystals on amorphous substrates, hindering their large-scale integration into functional devices. An advantageous alternative to obtain 1D nanostructures is to use a crystalline substrate for the growth, which induces the growth of the epilayer along preferred directions. This method, known as the guided growth approach,^20^ was extensively generalized by Joselevich and co-workers to a list of semiconductor nanowires, including heterostructures,^20−24^ enabling precise control over their crystallographic orientation, direction, and polarity.^25^ The unique in-plane growth mechanism was explored in many systems^26−28^ and supports epitaxial growth, driven by the minimization of interfacial energy, which is manifested as mismatch minimization between the substrate and the epilayer,^29^ leading in turns to the growth along specific directions and orientations. Another mode of guided growth is graphoepitaxy along nanometric features on the surface, such as nanosteps,^30^ where the nanosteps break the formation energy degeneracy, prompting a single favorable direction for the crystal nucleation.^31^ Graphoepitaxy has already been demonstrated as an efficient way to synthesize aligned TMDs 2D nanoflakes on vicinal *C*-plane sapphire.^32^

Vapor-phase synthesis of nanomaterials can involve different growth mechanisms, such as the catalytic vapor–liquid–solid (VLS) or the noncatalytic vapor–solid (VS), each producing intriguing structures from the bottom-up. One inspiring case is that of semiconductor nanofins, previously demonstrated for ZnTe,^33^ ZnO,^34^ and CdS nanowalls integrated into 3D nanodevices, also known as finFETs.^35^ In the last instance, the combination between the two mechanisms (VLS + VS) occurs at different rates, inducing the growth of high nanofin mixing features of 1D and 2D dimensionalities within the same nanostructure. Such growth of nanofins has great potential to extend Moor’s law with further miniaturization of the devices, allowing an increase in the device density per unit area.^34^ Following this approach, Intel presented in 2011^36^ the finFET, a transistor design based on a fin-like structure of the channel material. This new architecture enabled the transistors to scale down to 22 nm in 2012, all the way down to 3 nm, as reported by Samsung^37^ and TSMC.^38^ In the near future, persistent research of fin-channel-based devices is expected to extend the semiconductors market to further miniaturization, reaching “1.4 nm-technology”.^39^ In this context, the dangling-bond-free nature of 2D materials and their superior optical properties make layered van-der Waals materials potential components in high-performance fin-channel devices.^40^ Many efforts had been done to create vertical and aligned nanostructures of MoS_2_ in a controlled way, among them vertical nanosheets^41^ or triangles^42^ on SiC or out-of-plane vertical flakes and layers.^43,44^ Yet, full control over the dimensionality and alignment of the MoS_2_ vertical structure, as well as the study of their electronic and optoelectronic properties, is still lacking.

Herein, we report the space-confined CVD method to synthesize a unique mixed-dimensional 1D MoS_2_ we termed as “nanofin-nanoribbon hybrid”, which will sometimes be referred later as just “hybrid”. The hybrid nanostructures were grown epitaxially on a *C*-plane sapphire (0001) substrate, preferentially along the three isomorphic *m*- ± <11̅00> directions of the *C*-plane. We characterized the structural and optical properties of the hybrid by photoluminescence, cathodoluminescence, and Raman spectroscopy, showing a continuous structure with an optical response to visible light. We used the fin-like system to integrate the nanofin-nanoribbon hybrid into finFETs, showing their n-type characteristics with an on–off ratio of ∼10^3^ and charge carrier mobility of up to 0.4 cm V^–1^ s^–1^ at room temperature. The calculated charge carrier concentration is on the scale of ∼10^19^ cm^–3^. Our prototype device is a proof of concept for integrating a mixed-dimensional TMDs nanostructure into an operating finFET. Its performance is discussed in terms of crystal structure, purity, and device fabrication. Moreover, the hybrids were integrated into a fin-channel photodetector array, showing a fast response in the range of tens of microseconds and responsivity and detectivity comparable to previously reported values in similar MoS_2_-based PDs. We attribute the device’s performance to the high material crystallinity and the unique combination of dimensionalities in the hybrid structure.

## 2 Results and Discussion

### 2.1 Growth of MoS_2_ Nanofin-Nanoribbon Hybrids on *C*-Plane Sapphire

**Figure 1:**
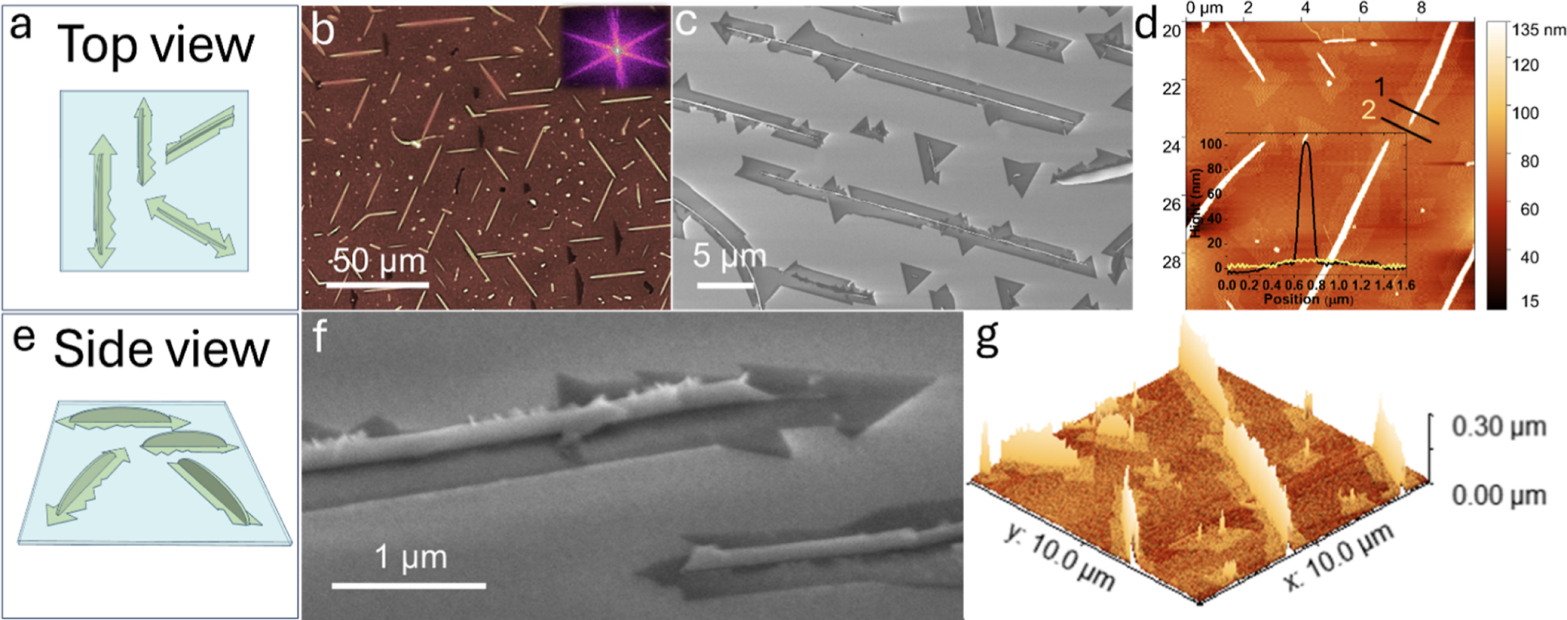
Structure and morphology of MoS_2_ nanofin-nanoribbon hybrid grown on *C*-plane sapphire. (a) Top-view schematics of a MoS_2_ nanofin-nanoribbon hybrid on *C*-plane sapphire. (b) Optical microscopy image capturing the MoS_2_ hybrid structure. The inset features a fast Fourier transform (FFT) pattern extracted from the image, highlighting the preferred three isomorphic orientations for growth. (c) A scanning electron microscopy (SEM) image shows the aligned MoS_2_ hybrid structures, where both nanoribbon and nanofin components are apparent. (d) Atomic force microscopy (AFM) scan of the MoS_2_ hybrid structure. The inset includes height line profiles 1 (black) for the nanofin part and 2 (yellow) for the nanoribbon part, indicating a height difference of approximately 100 nm between the two components. (e) Side-view schematics illustrating a MoS_2_ nanofin-nanoribbon hybrid on *C*-plane sapphire. (f) Side-view tilted SEM image showing the MoS_2_ hybrid structure. (g) 3D AFM image depicting multiple MoS_2_ hybrid structures on *C*-plane sapphire.

The MoS_2_ nanofin-nanoribbon hybrid was synthesized in a home-built CVD system within a microcavity-based configuration featuring two distinct heating zones. The full details appear in the experimental section, and the synthesis setup is schematically described in Figure S1. Briefly, a silica boat loaded with MoO_3_ powder is placed in a high-temperature zone (∼750 °C) with the *C*-plane substrate positioned on top, covered with a Si/SiO_2_ piece. This Si/SiO_2_ cover was utilized to enhance control over the deposition of the metal source in a manner similar to previously reported techniques.^10^ In parallel, another alumina crucible containing sulfur chunks was located upstream in the second heating zone (∼200 °C), serving as the source for the chalcogenide component. The symmetry fitting between MoS_2_ (C_3v_) and the *C*-plane sapphire^45^ allows the growth of MoS_2_ crystals with controlled directions and orientations dictated by epitaxy. Figure 1 provides an overview of the distinctive morphological characteristics resulting from the synthesis. Figure 1a presents the schematics of the resulting nanostructures, viewed from the top. In Figure 1b, we present an optical microscopy (OM) image of the sample revealing 1D-like structures with lengths ranging from 3 to 20 μm and 0D grains. A higher magnification optical microscopy image is presented in Figure S2(a), enabling the showing of a 2D nanoribbon decorating the central 1D structure. Notably, the fast Fourier transform (FFT), inset to Figure 1b, demonstrates six preferred directions for the 1D-structure growth, following the typical 3-fold symmetry of *C*-plane sapphire.^45^ We used the surface-sensitive “in-lens” SE detector in the scanning electron microscope to observe the 2D features in the sample. Top and tilted views of the structures are presented in Figure 1c,f, respectively.

The surface structures represent hybrids of two components displaying features of different dimensionalities within a single nanostructure. One morphology is a 1D structure that contains a thin, high fin-like structure that stands in the center and is decorated with a thinner, two-dimensional nanoribbon. This structure resembles the previously reported MoS_2_ flakes, which is also apparent in our samples. The flakes are characterized by a thick 0D nucleus at their center.^10,46^ This nucleation center is significantly thicker than the 2D flake, as measured from an atomic force microscopy (AFM) scan shown in Figure S2. In our case, the structure adopts a 1D dimensionality for both the thick core nucleus and thin decoration, which represents a mixed-dimensional nanomaterial of MoS_2_. The width of the nanoribbon component measures around 2 μm, with the edges displaying variation, appearing either serrated or straight and well-defined. Both edge shapes are evident in Figure 1c and with larger magnification in Figure 1f, signifying that the ribbon is formed by MoS_2_ flakes merging around the central high fin. We used AFM imaging to gain more information regarding the height of the different hybrid components. The results are presented in Figure 1d,g. Figure 1d shows a core 1D component, measuring a few hundred nanometers in height with an arc shape (line-profile 1). The 2D decoration consists of thin MoS_2_ flakes merging to form a few nanometers thick nanoribbon (line-profile 2), consistent with previously reported few-layered MoS_2_.^47^ To present the significant height disparity in this mixed-dimensional nanomaterial visually, an AFM 3D model of the hybrid structure is shown in Figure 1g. These structural features are further elucidated in the schematic representation of Figure 1e, showing a side-view profile of the sample.

**Figure 2:**
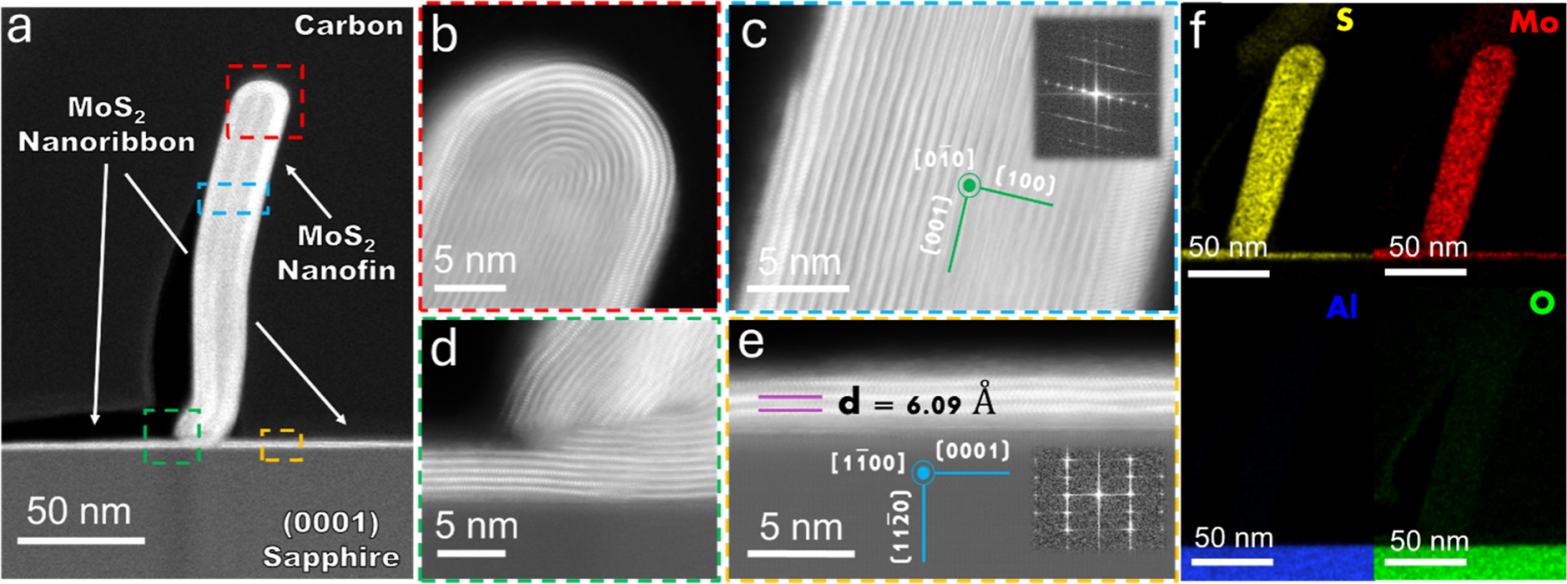
Structure of the MoS_2_ nanoribbon-nanofin hybrid. (a) Transmission electron microscopy (TEM) image of a cross-sectional sample capturing the MoS_2_ nanoribbon-nanofin hybrid growth on *C*-plane sapphire. White arrows indicate the nanofin and nanoribbon components. Different segments of the structure are highlighted in colored frames: top dome (red), nanofin body (blue), nanofin-nanoribbon interface (green), and nanoribbon (yellow). (b–d) are the corresponding higher magnification TEM images of the central dome, nanofin body, and nanofin-nanoribbon interface marked in red, blue, and green frames in (a), respectively. (e) TEM image displaying three-layered MoS_2_ nanoribbons as part of the hybrid structure (highlighted in (a) in a yellow rectangle). The measured *d*-spacing is 6.09 Å, aligning with previously reported values for MoS_2_ nanostructures. Insets feature fast Fourier transform (FFT) patterns indicating periodicity in the MoS_2_ crystal (c) and sapphire crystal (e) used to conclude the crystallographic orientations of the sapphire (e) and MoS_2_ (c). (f) Energy-dispersive X-ray spectroscopy (EDS) elemental maps of the hybrid structure, revealing sulfur (yellow) and Mo (red) content atop the aluminum (blue) and oxygen (green) elements of sapphire.

A study of the relationship between the nanoribbons and the central nanofin and an investigation of the epitaxial relations between the growing MoS_2_ hybrid nanostructures and the sapphire substrate were done by looking at its cross-section. A thin cross-sectional lamella was cut using a focused ion beam (FIB) followed by observation in a high-resolution transmission electron microscope. In Figure 2, we present characteristic findings of a typical hybrid nanostructure. Figure 2a shows a cross-sectional TEM image of a nanofin-nanoribbon hybrid, underscoring the significant height contrast between the two primary components, the nanofin and the nanoribbon, as indicated by the arrows. To offer a more detailed view, we highlight four distinct subparts of the structure with colored frames in Figure 2: a rounded dome shape at the top of the nanofin (red), the main body of the nanofin (blue), the nanofin-nanoribbon interface (green), and the nanoribbon itself (yellow).

For a closer examination of these individual components, we provide higher magnification images in Figure 2b–e. Throughout these images, the characteristic layered van der Waals structure of MoS_2_ is evident in all parts of the hybrid structure. The nanofin emerges as a multilayered structure with layers stacked vertically relative to the substrate. At the same time, the nanoribbon exhibits a few-layered MoS_2_ arrangement oriented horizontally to the substrate parallel to the (0001) planes of sapphire. Notably, the layer-to-layer distance was measured to be approximately 6 Å, consistent with previous observations for 2H–MoS_2_.^47^ Based on our crystallographic analysis of the sapphire and the well-known 3-fold symmetry of *C*-plane sapphire, we deduced that the growth of the 1D nanofin-nanoribbon hybrid structure occurs in alignment with the three isomorphic *m*, ⟨11̅00⟩, directions of the *C*-surface of sapphire. In Figure 2f, we present elemental energy-dispersive X-ray spectroscopy (EDS) maps that illustrate the distribution of molybdenum and sulfur within the 1D hybrid structure, along with the presence of aluminum and oxygen components from the sapphire substrates. A quantitative analysis of the EDS signals for a typical hybrid, as presented in Figure S3, reveals the expected Mo/S ratio of ∼1:2. It is worth mentioning that the stoichiometric ratio in some of the hybrids frequently points to an excess of Mo atoms, which can be attributed to S vacancies in the structure. To gain more accurate understanding of the stoichiometric ratio in our MoS_2_ hybrid sample, we conducted an X-ray photoelectron spectroscopy (XPS) measurement on an area of the sample with high deposition level. Conducting measurements on such an area helps us to gain an insight into a large hybrid ensemble. XPS spectra and detailed values are shown in Figure S4. Our MoS_2_ nanofin-nanoribbon hybrids exhibit the Mo doublets 3d_5/2_ and 3d_3/2_ at 229.8 and 232.9 eV, as reported for Mo^4+^ of MoS_2_.^48^ This spectral range also contains the S-2s peak at 227 eV, in agreement with S in MoS_2_. The main S-2p doublets at 162.9 and 164 eV follow reported values S 2p_3/2_ and 2p_1/2_ of MoS_2_. The Mo 3d area has a low binding energy shoulder (232.9 eV, 236 eV) corresponding to Mo^6+^, attributed to the residual MoO_3_ source material on the surface.^48^ However, our TEM-EDS measurements and mapping do not show detectable oxide impurity, suggesting that this contamination is external to the MoS_2_ hybrids, for example, absorbed on top of the sapphire substrate. XPS-derived concentrations yield a nominal range of 1.91:1 to 2:1 ratio for S: Mo, after excluding ∼20% of Mo intensity attributed to MoO_3_. Relying on TEM-EDS results and the XPS measured ratio, we ruled out that sulfur vacancies are probably introduced into the crystal.

#### 2.1.1 Growth Model

**Figure 3:**
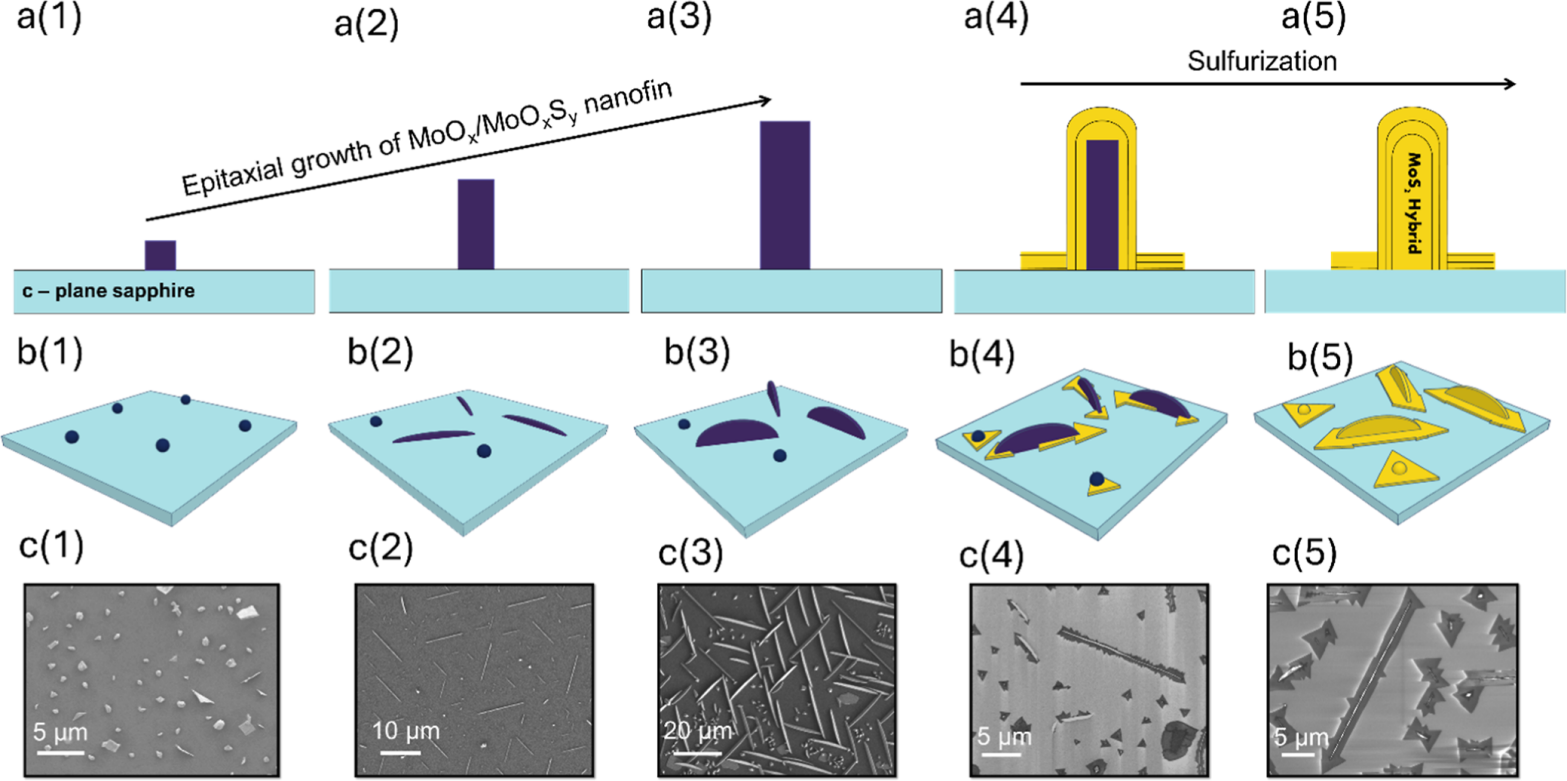
Growth model of the nanofin-nanoribbon hybrid. The growth process unfolds in two distinct stages. In the initial stage, there is the nucleation and epitaxial growth of molybdenum suboxide particles and nanowires, eventually evolving into a nanofin as the synthesis proceeds. Schematics in a(1–3) represent the nanostructure cross-section and in b(1–3) represent a top view of the substrate. The corresponding SEM images are shown in c(1–3). Upon the introduction of higher sulfur flux into the system during the second stage, gradual sulfurization takes place, resulting in the formation of a MoS_2_ nanofin-nanoribbon hybrid, as shown in the schematics in a(4–5) and b(4–5) and the corresponding SEM images in c(4–5).

The conventional two-step synthesis of MoS_2_ involves the initial step of heating and decomposing MoO_3_. At this stage, MoO_3_ nucleates and grows on the substrate, a self-seeded growth that was reported before in CVD-grown MoS_2_.^10,49^ In some cases, a crystallographic relation could be recognized between the preliminary deposited oxide and the later growing MoS_2_ epilayer, while the predeposited oxide sets many times the growth direction of the MoS_2_ with respect to the substrate.^50^ The growth of core–shell MoO_2_@MoS_2_ was reported before^51^ and followed the same stages of synthesis we apply here. In that case, the central structure was nanorods of MoO_2_, which went over gradual sulfurization from the outside inward, creating a thin MoS_2_ shell at the gas–solid interface. We suggest that the MoS_2_ nanofin-nanoribbon hybrids are formed gradually over the two phases of the process, as illustrated in Figure 3. In this figure, purple structures denote MoO_*x*_ structures, while yellow denotes MoS_2_ features. The figure shows, with different sequential schemes, the gradual formation of the MoS_2_ nanofin-nanoribbon hybrid as we suggest here (Figure 3a(1–5) for the hybrid nanostructure cross-section and Figure 3b(1–5) for the hybrid nanostructure from top). The promotion of the process supports the model, as it was documented using SEM (Figure 3c(1–5)) over various samples with different sulfurization conditions. All SEM images were taken during the process of optimization for conditions to grow the hybridss. Figure 3c(1–3) represents a sample with a short overall one-step reaction time, where MoO_3_ and S are heated together. In Figure 3c(4), we present a sample with a gap in the heating time of S with respect to MoO_3_, that is, separating the process into two distinct stages, and in Figure 3c(5), we show a sample with similar synthesis but with longer sulfurization time and higher gas flow rate, yielding a fully sulfurized nanofin-nanoribbon hybrid. The synthesis starts with MoO_*x*_ nucleation and epitaxial growth on the sapphire surfaces, as evidenced by the controlled orientations observed in the *C*-plane. It was pointed before that a central parameter controlling the growth of MoS_2_ is the ratio between the reactants, specifically the S to Mo source ratio, denoted as S/Mo.^49^ A higher concentration of the reactants promotes self-seeding nucleation, resulting in the formation of 0D nucleation sites. These 0D nucleation sites are evident in our AFM (Figures 1 and S2) and SEM image (Figure 3c(1)). These 0D sites subsequently merge and elongate into a 1D structure that will, followingly, turn into a multilayered nanofin, as illustrated in Figure 3(4–5). The second phase of the process starts when S vapors are delivered. The sulfurization of the nanofins occurs gradually according to the following overall reaction:^52^
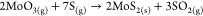


The detailed mechanism for this reaction often involves the creation of MoO_3–*x*_ and MoO_3–*x*_S_*y*_ intermediates at the early stage structure found on the substrates.^52^ Evidence for the existence of such suboxide phases in our system was observed when the same synthesis conditions were applied to a different sapphire surface: *A*-plane sapphire. We found out that the synthesis of nanofin-nanoribbon on *A*-plane sapphire exhibited limited sulfurization under the same conditions, retaining a distinct MoO_3–*x*_ phase at the core of the nanostructure. Figure S5 presents the primary structural features of the nanofins grown on *A*-plane sapphire. Specifically, Figure S5c shows a cross-sectional EDS map of a single nanofin, revealing a core of MoO_3–*x*_ and a shell of MoS_2_. These findings further support the model we suggest here regarding the gradual sulfurization of predeposited structures of MoO_3–*x*_. The sulfurization process progresses gradually. At lower sulfur-zone temperatures (200°) and lower gas flow rates (82 sccm N_2_), discontinuous nanoribbons with serrated edges are formed. As noted earlier, the S/Mo ratio plays an important role in determining the morphology of the resulting MoS_2_ nanostructures. A fundamental step in the formation of lateral structures is related to the predeposited MoO_*x*_ crystals,^44^ which are strongly influenced by the S/Mo ratio. Higher S/Mo ratios promote more homogeneous growth primarily at the solid–gas interface.^53^ This homogeneous growth favors the lateral growth of the predeposited MoO_*x*_, commonly yielding standing flakes that will subsequently react with the chalcogen (S) vapors. By controlling the reactants ratio in the reaction tube, we observed a transition to a more continuous nanoribbon formation. Increasing the sulfur-zone temperature to 270° and the gas flow rate to ∼90 sccm enhanced the S/Mo ratio, which optimized the conditions for lateral growth of MoO_*x*_ fins, as well as their full sulfurization. This also resulted in the formation of well-defined edges for the nanoribbons decorating the central fin. These outcomes are illustrated schematically in Figure 3(4–5) and in the SEM images in Figure 3(4–5).

To generalize the guided growth of the MoS_2_ nanofin-nanoribbon hybrid and further examine the suggested mechanism for creating mixed-dimensional nanofins of MoS_2_, we applied the growth process to a faceted substrate, manifesting a different mode of guided growth referred to as graphoepitaxy.^20^ In graphoepitaxy, the substrate exposes different facets that spontaneously form on the surface and, in turn, create guiding nanofeatures on the surface. One well-studied case is that of *M*-plane (101̅0) sapphire, which is thermodynamically unstable^54^ and undergoes spontaneous faceting upon being annealed in air at high temperatures (1600 °C). The more stable *S* (101̅1) and *R* (11̅02) facets are exposed and create V-shaped nanogrooves oriented in the *A* direction, ± [12̅10], of sapphire crystal. The depth and width, as well as the nanogroove pitch, are being set by the annealing temperature. In that case, the guided growth of the nanostructure is dictated by the graphoepitaxial effect, driven by minimizing the surface energy of the nanostructure, promoting a surface adjacent growth of the nanostructure as observed previously for various II–VI semiconductor nanowires.^20,22,23,55^ When MoS_2_ is grown on annealed *M*-plane sapphire, it forms well-aligned nanofins typically leaning on one of the facets of the nanogroove. The structural properties of the nanofins grown on the annealed *M*-plane are presented in Figure S6. Figure S6a shows a typical SEM image of epitaxially oriented MoS_2_ nanofins grown on an annealed *M*-plane. The higher magnification SEM image shows a single nanofin. The nanofins range from 2 to 6 μm in length, lacking the characteristic nanoribbon part observed in the case of *C*-plane-grown hybrids. The height of the nanofins was measured and imaged using AFM; the results are summarized in Figures S6b,c. The height ranges from a few tens of nanometers, aligning with the multilayered appearance of the nanofins grown on the *C*-plane. Raman spectroscopy confirmed the crystal structure of the nanofins, detecting the characteristic A_1g_ and E_2g_ Raman modes of MoS_2_, as shown in Figure S6d. The cross-section of the nanofins was observed by high-resolution transmission electron microscopy (HR-TEM), and the results are summarized in Figure S7. Figure S7a shows a typical nanofin grown inside a nanogroove of an annealed *M*-plane sapphire. The same nanofin is shown with a higher magnification in Figure S7b. The edge of the nanofin highlighted in Figure S7b with a red square and the center of it, highlighted in Figure S7b with a light blue rectangle, are shown in Figure S7c,f with higher magnification, respectively. Although resembling a multilayered nanoribbon since it is not vertically standing with respect to the sapphire *M*-plane, we relate the nanostructure as a nanofin as it is a free-standing nanostructure exposing, from a certain point, also its bottom surface. The chemical composition of the nanofins was examined using EDS, and the elemental maps are presented in Figure S7d,e, showing molybdenum–aluminum and sulfur–oxygen maps, respectively. The S/Mo ratio in the nanofin is approximately 2:1, aligning with the MoS_2_ chemical composition. The growth of MoS_2_ nanofins on annealed *M*-plane sapphire generalizes the growth of this mixed-dimensional nanostructure to another mode of guided growth. It allows an additional degree of freedom to control the orientation of this structure. The growth of nanofins on annealed *M*-plane sapphire also supports the mechanism presented here. The tendency of MoO_*x*_ nanowires to grow high to create nanofins and their following sulfurization while preserving the original fin dimensions yield a fin-like structure of MoS_2_.

### 2.2 Optical Characterization

**Figure 4:**
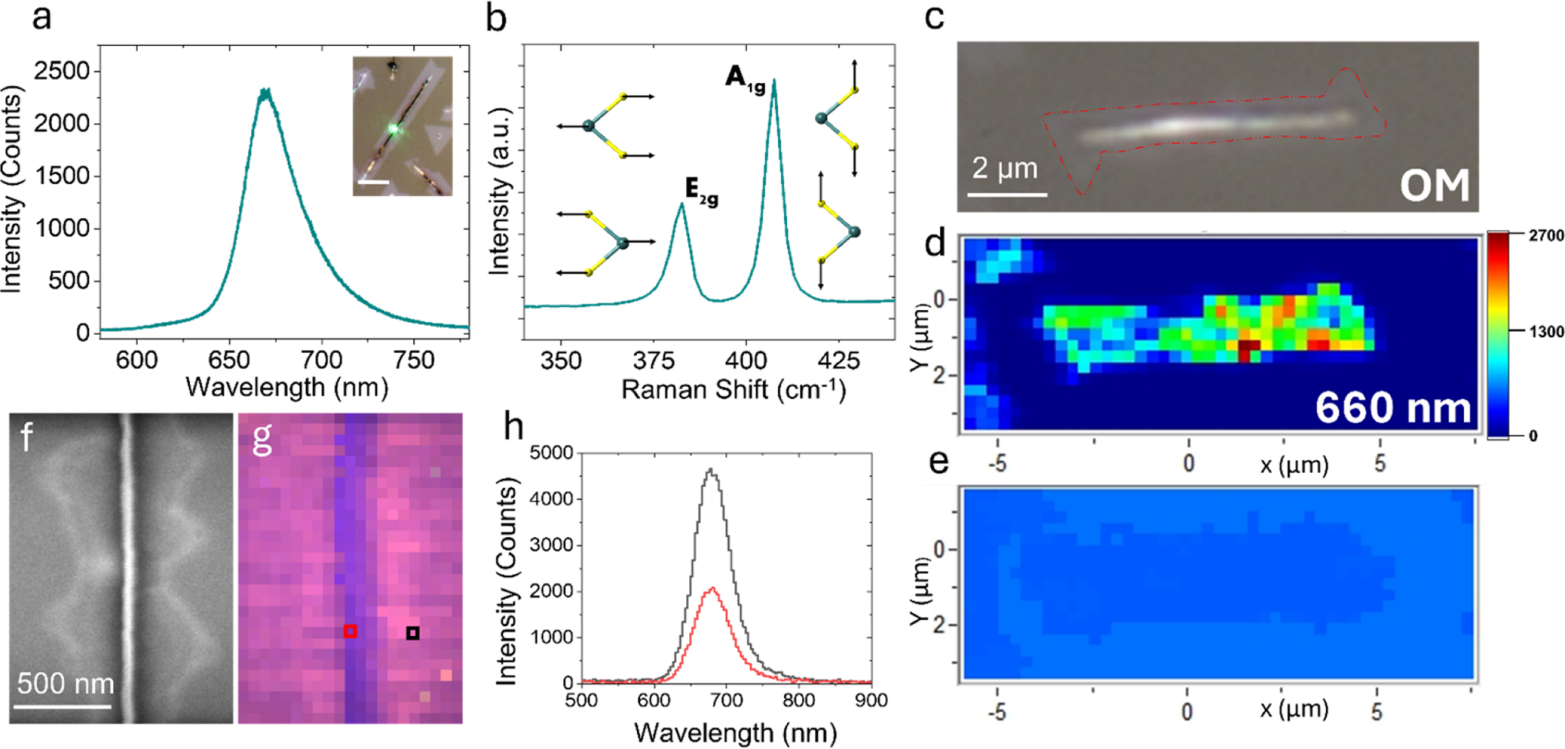
Optical properties of the MoS_2_ nanofin-nanoribbon hybrid. (a) The photoluminescence (PL) spectrum of an individual MoS_2_ nanofin-nanoribbon hybrid grown on *C*-plane sapphire was recorded using a 532 nm excitation laser. Inset is an OM image showing the exact position from which PL and Raman spectra were collected and are shown in (a) and (b); scale bar is 1 μm. (b) The Raman spectrum of a single MoS_2_ nanofin-nanoribbon hybrid grown on *C*-plane sapphire was obtained with a 532 nm excitation laser. It reveals the two-characteristic high-frequency modes: A_1g_ at ∼407 cm^–1^ and E_2g_ at ∼382 cm^–1^, demonstrating a 25 cm^–1^ difference, consistent with previous reports on multilayered MoS_2_. (c–e) Corresponding optical microscopy images (the red line is a guide to the eye for nanoribbon edges), a PL intensity map at 660 nm, and a PL peak position map of a single nanofin-nanoribbon hybrid nanostructure, respectively. (f) SEM image of a single nanofin-nanoribbon hybrid nanostructure. (g) A cathodoluminescence (CL) real-color intensity map of the same MoS_2_ hybrid structure is presented in (f). (h) Site-specific CL spectra relating to the map data in (g). The black and red lines correspond to the pixels marked in black and red in the map.

To further investigate the thickness and chemical composition of the nanofin-nanoribbon system, we conducted additional examinations through optical properties, including room-temperature Raman spectroscopy, photoluminescence (PL), and cathodoluminescence (CL) measurements of the hybrid structure. A summary of the results is provided in Figure 4. Figure 4a shows the hybrid structure’s photoluminescence (PL) spectrum, which was conducted with the excitation of a 532 nm green laser (frequency double Nd/YAG). The PL signal is predominantly centered around the 660–680 nm range, indicative of a band-edge emission at approximately 1.8 eV, in agreement with previously reported values for MoS_2_.^10^ Although they have a multilayered MoS_2_ structure (both nanoribbon and nanofin), the hybrids exhibit high PL intensity. This kind of high-intensity luminescence was observed before in multilayered MoS_2_ and can be related to strain existing in the structure, mainly around the curved area at the nanofin-nanoribbon interface.^50^ The deviation in band edge emission with respect to bulk MoS_2_, covering a range of values, may be explained by strain induced into the structure due to the epitaxial growth or the Burstein–Moss effect, as previously shown for ZnS nanowires.^23^ This effect is known to exist under conditions where the semiconductor is highly doped. The charge carrier distribution is discussed in detail in the Electrical Characterization section.^56^ In addition to PL measurements, the Raman spectrum was collected from the MoS_2_ nanofin-nanoribbon hybrid, with the same laser line, showing the characteristic Raman modes^57^ E_2g_^1^ and A_1g_ at ∼383 cm^–1^ and 407 cm^–1^, respectively. The E_2g_^1^ to A_1g_ peak-to-peak separation indicates the crystal thickness and, more accurately, the number of MoS_2_ layers stacked together. Upon a higher number of stacked layers, the A_1g_ peak moves to higher frequencies due to the increased vdW force, which suppresses atom vibrations and results in higher force constants. On the contrary, the E_2g_^1^ moves to lower frequencies, a shift that is related to long-range Coulombic interactions in multilayered MoS_2_.^58^ This separation was measured to be ∼25 cm^–1^, usually identified with multilayered MoS_2_.^59^ The optical characterization goes in line with our TEM and AFM information regarding the few multilayered MoS_2_ hybrid structures that were inferred.

We conducted comprehensive PL and CL mapping experiments to gain insight into the interplay between the nanoribbon and nanofin and their respective roles in the light–matter interaction within this mixed-dimensional nanostructure. The initial mapping experiment is depicted in Figure 4c through 4e. Figure 4c showcases an optical microscopy image of a single nanoribbon-nanofin hybrid, and the red line guides the eye toward the nanoribbon component edges. We subsequently performed PL mapping of a single MoS_2_ hybrid by illuminating the sample with a 532 nm green laser and mapping the resulting emission intensity at 660 nm. The PL intensity map is presented in Figure 4d. While this map’s resolution may not be sufficient to distinctly point at emission variations between the nanofin and the nanoribbon, it reveals that thicker regions of the structure exhibit weaker luminescence compared to their thinner counterparts. It is worth noting that sample thickness can influence emission intensity.^2^ Still, the distribution of peak positions (wavelength of the emission peak center), as shown in the peak-position map in Figure 4e, remains notably narrow and unchanged. This observation underscores the high uniformity of the emission, reflecting the overall consistency of the MoS_2_ crystal throughout the entire structure.

An additional mapping experiment was done on a different nanofin-nanoribbon hybrid by utilizing a cathodoluminescence measurement system in the scanning electron microscope. The findings of the cathodoluminescence (CL) mapping are summarized in Figure 4f through 4h. Figure 4f displays a scanning electron microscopy image of a nanofin-nanoribbon hybrid captured by using a photomultiplier tube (PMT) detector. Figure 4g presents a real-color CL intensity map highlighting the luminescence peak at approximately 680 nm from the same structure. Unlike PL mapping, the resolution in CL mapping is sufficiently high to reveal a pronounced, distinct intensity contrast between the core nanofin and the decorating nanoribbon. To provide a more precise visualization of this disparity, we selected sampling points from the nanofin (indicated by the red square in Figure 4g) and the nanoribbon (indicated by the black square in Figure 4g) and plotted the corresponding CL spectra. Figure 4h presents the CL spectra, with the black line representing the nanoribbon’s emission and the red line representing the nanofin’s emission. These spectra align with the PL mapping information, further confirming that the primary optical response intensity of the material is associated with its thinner part, the nanoribbon. The emission remains centered at approximately 1.8 eV, with no significant deviations observed among the different components. The weak luminescence of the thick nanofin part and uniform band-edge emission position across the entire hybrid could also be explained by the primary excitons created in the thinner, few-layered nanoribbons, which later can migrate due to the changing band-alignments across the hybrid^8^ and luminesce in the nanofin part.^2,60^ The migration becomes even more pronounced under the high excitation energy available in the CL measurement, inducing hot carriers into the system.

### 2.3 Electrical Characterization

**Figure 5:**
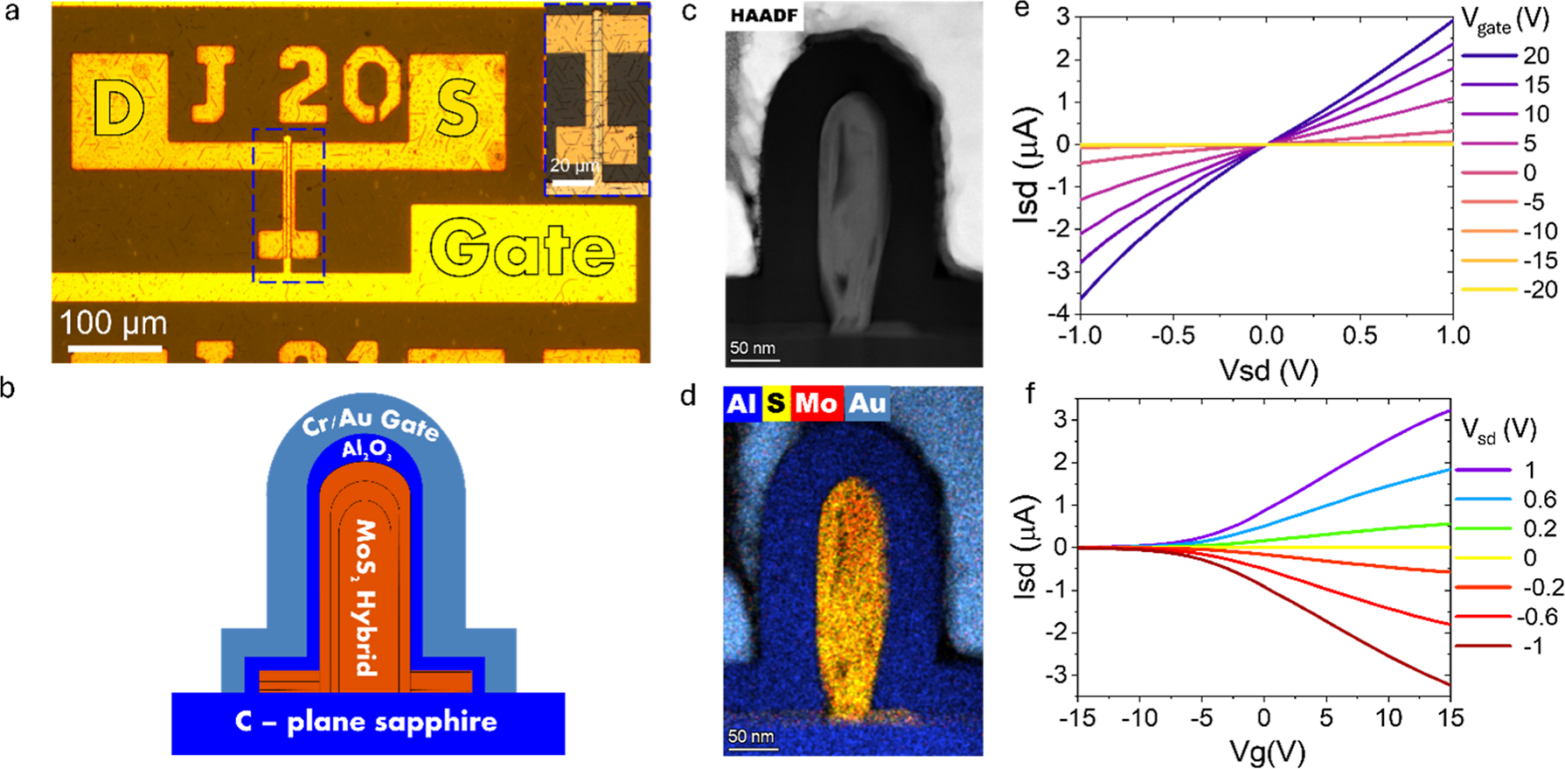
Performance of a MoS_2_ nanofin-nanoribbon hybrid-based finFET. (a) OM image of MoS_2_ hybrid-based finFET. Source, drain, and gate electrodes are shown. Inset is a larger magnification OM image of the device channel, shown in the image with a blue rectangle. (b) Model of the finFET cross-section showing the layers in the device, including the MoS_2_ hybrid as the channel material (orange), Al_2_O_3_ as the dielectric (blue), and a Cr/Au gate electrode (pale blue). (c) Cross-section dark-field TEM image of a single MoS_2_ nanofin-nanoribbon hybrid in a typical finFET channel. (d) An EDS elemental map of the same hybrid is presented in (c), showing the aluminum (blue), sulfur (yellow), molybdenum (red), and gold (pale blue) content along the different layers of the device. (e) Characteristic *I*_sd_ vs *V*_sd_ recorded for different *V*_g_. (f) *I*_sd_ vs. *V*_g_ at different source-drain bias; the pseudolinear part’s slope is used to calculate the transconductance, *g*_m_.

The electrical properties of the MoS_2_ nanofin-nanoribbon hybrids were investigated by fabricating a prototype finFET with epitaxially oriented MoS_2_ hybrids bridging two source-drain electrodes and a top gate. The guided growth approach provides control over the growth directions of the MoS_2_ hybrids. This directional growth allows the alignment of the hybrids in the three preferred orientations. Upon choosing a certain direction out of the three available, several parallel hybrids, bridging the source drain electrodes, can be measured. The fabrication was done using standard photolithography and electron-beam evaporation for the deposition of Cr/Au (25/250 nm) for the source-drain electrodes with a 5 μm gap. To ensure proper adhesion of the conformal Al_2_O_3_ layer to the nanofins, a thin layer of aluminum (Al) of about 1 nm was deposited on the nanofins, prior to an atomic layer deposition (ALD) process. This layer is expected to oxidize quickly to Al_2_O_3_, promoting the thicker, ALD-deposited Al_2_O_3_ dielectric layer. Followed by this stage, a 50 nm Al_2_O_3_ dielectric layer was deposited using standard atomic layer deposition. The gate electrode was fabricated using a similar photolithography step, followed by angle evaporation of Cr and Au. The sample was positioned on a 45° tilted stage for deposition of Cr/Au (10/100 nm), followed by a 180° rotation for another cycle of Cr/Au (10/100 nm). Finally, an additional 100 nm of Au was deposited on top. These stages ensure that the gate electrode fits the fin-like dimensions of the hybrid. The measurements were conducted under a vacuum (∼10^–5^ bar) and a room-temperature atmosphere. Figure 5 shows a typical device and its performance. In Figure 5a, a true color OM image shows with low-magnification the two-terminal device with a top gate and a high magnification image of the device channel. Figure 5b presents a cross-sectional model of the device, illustrating the layers comprising MoS_2_ hybrids as the channel material (orange), Al_2_O_3_ as the dielectric (blue), and Cr/Au electrode serving as a top gate (pale blue). To verify that the fabrication yielded a trigate field-effect transistor, we cut a thin lamella of the device’s channel cross-section utilizing the fin dimensions. A typical cross-section of a MoS_2_ hybrid within a device is shown in a dark-field image and with an EDS elemental map in Figure 5c,d, respectively. The elemental mapping in Figure 5d exhibits the device’s different layers and the trigating of the fin-channel. Figure 5e shows a two-terminal electrical measurement done by biasing the device within the range of −1 V to 1 V under different gate voltages. The linearity of the curves at low source-drain voltage suggests ohmic contacts within the device. The current increase with forward bias indicates the well-known n-type character of the MoS_2_ channel.^57^ In Figure 5f, the presented *I*_sd_ vs *V*_g_ at different bias voltages further shows the n-type character of the channel with a threshold voltage of *V*_th_ ∼ −15 V and an on–off ratio up to ∼10^3^. The slope of the linear part of this curve is used to estimate the transconductance, *g*_m_, which in turn is used to calculate the charge carrier mobility in the device according to eq 1. Here, *L* is the channel length and *C*_0_ denotes the dielectric layer capacitance, calculated using the assumption of the quasi-rectangular cross-section as shown in eq 2. Herein, ε and ε_0_ are the dielectric layer and vacuum permittivity constants, respectively. *h* signifies the nanofin height, *w*_1_ and *w*_2_ are the nanofin and nanoribbon widths, respectively, *d* is the dielectric layer thickness, *L* is the channel length, and *n* is the number of hybrids in the channel. The calculated room-temperature mobility was in the range of 0.08–0.4 cm^2^ V^–1^ s^–1^. The carrier concentration was calculated using the threshold voltage, *V*_th_, and is in the order of 10^19^ cm^–3^. The high charge carrier concentration goes in line with the threshold voltage observed here, as a higher electron concentration typically requires more negative values to fully close the channel. Such a threshold voltage may be explained by the presence of point defects, as S vacancies induce n-type doping into the structure, as we see in the nonperfect stoichiometric ratio in the EDS measurements and was discussed in our XPS measurements, that points at the sulfur-deficient structure. This can also account for the recorded low mobility range as the vacancies act as scattering sites.^61^
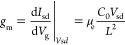
1
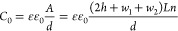
2
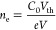
3

It is worth noting that the calculated values for electron mobility and concentration indicate that the high electron concentration exceeds the noninteracting limit,^62^ which can make electron–electron scattering the dominant mechanism, effectively reducing the mobility. This trade-off, where increased charge carrier concentration enhances conductivity but is counterbalanced by scattering from point defects and electron–electron interactions, can result in the mobility values observed here.^63^ In addition, it has been previously demonstrated that defects on the surface, typically scale roughly linearly with the nanostructures dimension (radius) in the channel,^21,64^ as well as significant phonon scattering at room-temperature^65^ can lead to a considerable drop in measured mobility within the device by orders of magnitude. We note that in some of the fins, a layer separation that induces voids into the structure is observed. This phenomenon was observed partially in our TEM results, as depicted in Figure 5c, and may influence the way the electric field is applied across the channel. This occurs randomly in some of the hybrids and is assumed to imply the device’s performance. Suggestions for improving our device performance may involve employing high-κ dielectric^66^ and improved contacts to lower resistivity. Another option for mitigating the influence of vacancies is further annealing under a sulfur atmosphere. Despite the room temperature mobility values being somewhat lower than previously reported values for similar devices based on MoS_2_,^47,57^ our device exhibits a relatively high on–off ratio and the n-type character of MoS_2_ well. This preliminary device serves as a proof of concept, pioneering the integration of TMDs into a room-temperature operating finFET.

### 2.4 Optoelectronic Characterization

**Figure 6:**
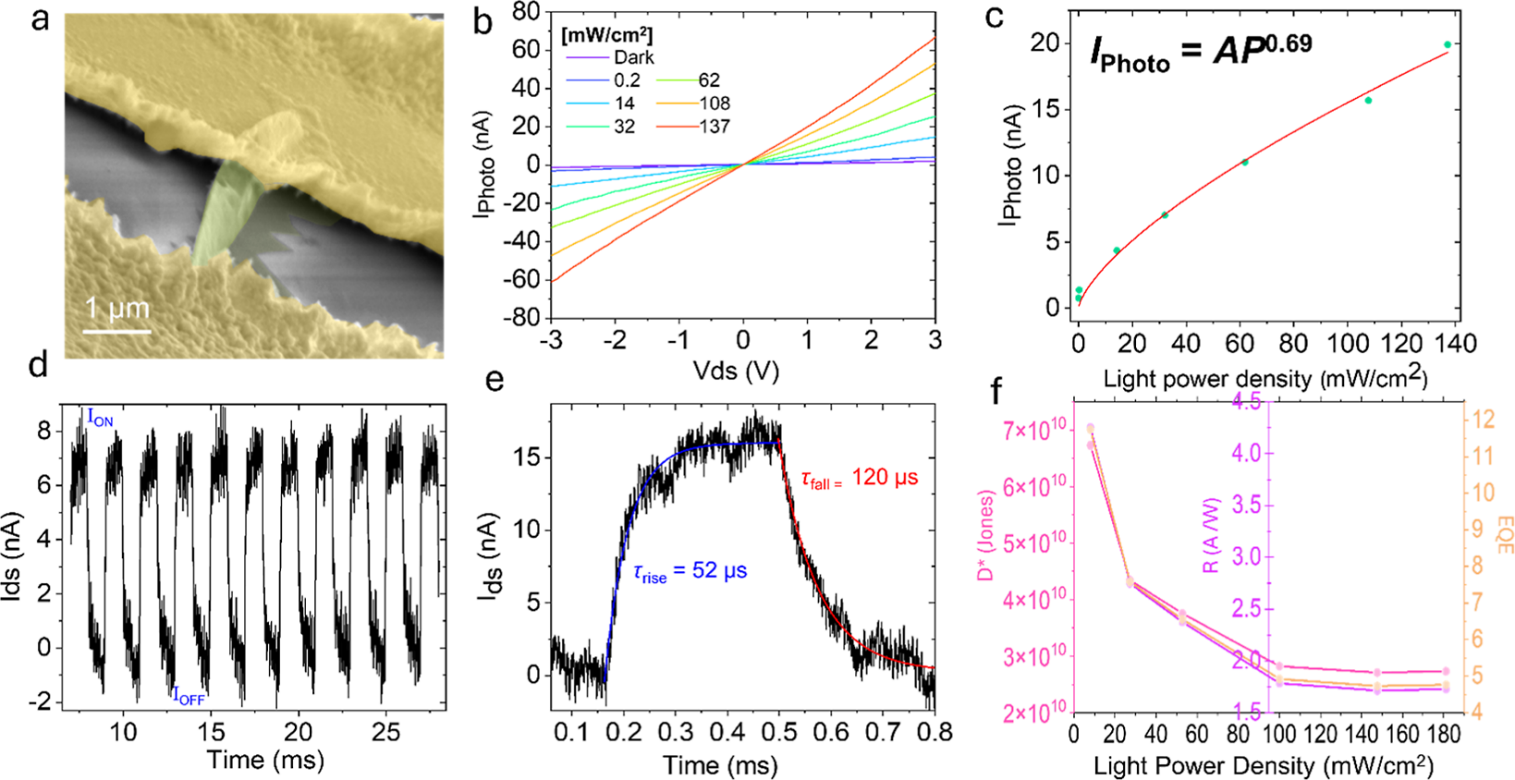
Optoelectronic characterization of the MoS_2_ nanoribbon-nanofin hybrid. (a) False-colored tilted SEM image of the hybrid structure (green) bridging the two source-drain electrodes (gold). (b) *I*–*V* curves measured from a typical photodetector under sweeping voltage in the range of −3 to 3 V and increasing 450 nm laser power densities at 0 (dark) to ∼137 mW cm^–2^. (c) A typical exponential fit of the photocurrent as a function of laser power density yields the relation *I* = *AP*^0.69^. (d) Dynamic photocurrent modulations in a typical photodetector under 11 on–off switching cycles done using an acousto-optic modulator (AOM) shifting the beam on and off the device with a frequency of 500 Hz. (10 V bias and 8.5 mW cm^–2^). (e) Single on–off switching cycle with higher resolution. Single exponential fitting curves to the rise and fall stages of the current are presented in blue and red, respectively, yielding response times of 52 and 120 μs, respectively. (f) Responsivity, detectivity, and external quantum efficiency (EQE) changes with laser power density, in a typical MoS_2_ hybrid-based PD, yielding a characteristic decay.

The exceptional optical response of the hybrid structure is a pivotal property for its integration into parallel photodetector arrays. This hybrid structure serves as the channel material in optoelectronic devices, specifically as an active element in photodetectors. The device was fabricated as a two-terminal photodetector array, with the MoS_2_ hybrid 1D nanostructure grown on a *C*-plane sapphire substrate. Each device comprises several hybrids connecting two Cr/Au (25/250 nm) source-drain electrodes with a 3 μm gap. In Figure 6a, we present the hybrid structure spanning the source-drain electrodes in a false-color, tilted SEM image, with the increased magnification highlighting the total structural characteristic of the device with a fin channel. To assess the photoresponse to visible light, the device was illuminated with a 450 nm laser and the photoresponse was examined under various laser power densities, including in the absence of illumination (dark conditions). This experiment was conducted while applying bias voltage sweeps ranging from −3 V to 3 V. The resulting *I*–*V* curves, depicting the photocurrent response to increasing light intensities, are illustrated in Figure 6b. The anticipated increase in current resulting from the increasing laser power densities is evident through the notably high ratio between the photocurrent and dark current, often termed the on/off ratio of the photodetector. This on/off ratio typically falls within the range of 8 to 35 at a 3 V bias and under maximal laser power density (∼100 mW cm^–2^). We attribute this on/off ratio to the large amount of channel material available to absorb a larger fraction of the incident light due to the thick and high nanofin,^60^ increasing the light to current conversion. Variations observed between different devices can be attributed to the distinct number of hybrids bridging each device and variations in their fine structure, particularly in terms of thickness and height, which were not uniform. The behavior of the photocurrent, along with its augmentation with increasing light intensities, is frequently described through a straightforward power law denoted as *I*_photo_ = *AP*^θ^. This widely used function yields a pre-exponential coefficient, *A*, dependent on the illumination wavelength, and a power exponent, θ, closely associated with the charge carrier lifetime.^21^ Our fitted results, presented in Figure 6c, yield a sublinear value of θ = 0.69 for the MoS_2_ hybrids. θ values spanning the range of 0.5 to 1.0 signify the presence of bound states distributed in a manner where their concentration diminishes as one moves farther away from the band edges. Albert Rose^67^ well-explained this behavior and proposed that the lifetime of free charge carriers decreases as light intensity increases. Thermal equilibrium is disrupted when the semiconductor undergoes excitation, creating two quasi-Fermi levels: one for electrons and one for holes. When the semiconductor is exposed to higher laser power densities, these quasi-Fermi levels shift toward their respective band edges, resulting in additional ground states. These newly created states act as recombination centers and come at the cost of shallow traps. Consequently, the heightened concentration of recombination centers leads to a reduction in the carrier lifetime. This phenomenon is reflected in the sublinear relationship between the current (*I*) and the laser power density (*P*). Other figures of merit characterizing the device are the responsivity (*R*_λ_), detectivity (D*), and the external quantum efficiency (EQE). Responsivity quantifies the current generated per incident light-power density unit and the effective illuminated device area. Responsivity is considered as a parameter to evaluate the efficiency of the photodetector with respect to photon-to-current transformation. It is calculated following eq 4
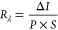
4In this equation, Δ*I* represents the net current resulting from light illumination (*I*_photo_ – *I*_dark_), *P* stands for the laser power density, and *S* denotes the effective area of the illuminated device. In our context, we estimate S by multiplying the electrode gap by the hybrid width and the number of hybrids connecting the electrodes. Detectivity is one of several parameters used to characterize the sensitivity of the device’s detection capabilities concerning the noise equivalent power. It is determined according to eq 5
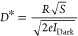
5

In the equation above, *R* represents the responsivity, *S* the illuminated channel area, *e* the elementary charge of an electron, and *I*_Dark_ the dark current. Last, the EQE is usually used to measure the capacity of the PD in transforming light to electrical charge carrier in the device and is frequently used to describe a photodetector performance. The EQE can be calculated according to eq 6
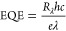
6Here, *R*_λ_ is the responsivity, *h* is the Planck constant, *c* is the speed of light, *e* is the elementary charge of the electron, and λ is the incident light wavelength. Figure 6f shows a typical trend of the mentioned figures of merit, decreasing with the light power density. In our measurements across five devices, the responsivity varied from 4.3 to 280 A W^–1^ under the lowest light power density and decreased to 3 A W^–1^ at the highest light power density. In order to have a better understanding of the responsivity to light power density relation, and specifically the decay trend, we fitted the responsivity data with a single-exponential function corresponding to . Here, *R*_λ_ is the responsivity, *R*_λ,P_max_ is the saturation value for the responsivity high light power densities, *A* is a pre-exponential constant signifying the maximal growth in *R*_λ_ with respect to *R*_λ,P_max_ as *P*→0, and π is the characteristic decay constant, representing the light power density leads to 1/*e* of the total decay. In our case, *R*_λ,P_max_ ranges from 1.7 to 3.4 A W^–1^ and with a typical decay constant π, ranging from 1.3 to 25.6 mW cm^–2^. The detectivity ranged from 3.81 × 10^12^ Jones at the lowest light-power density to 2.73 × 10^10^ Jones at the highest light-power density. Correspondingly, the EQE ranged from 794 at the lowest light power density to 4.7 at the highest. The calculated values are in line with previously reported photodetectors based on pristine MoS_2_ or its heterostructures.^68^ The model we proposed for the shortened charge carrier lifetime, as discussed earlier, can also elucidate the decline in responsivity with an increase in laser power density, as depicted in Figure 6f. As the detectivity and EQE are directly related to the responsivity, the later two adopt a similar trend and decrease with higher light power density. Furthermore, the device’s response time is a crucial parameter reflecting its performance, both in fundamental and practical terms. Various applications often necessitate swift device responses, and, parallelly, the response time is instrumental in comprehending the charge carrier dynamics in the channel material. To assess the device’s photoresponse, we subjected it to modulated 450 nm laser beam illumination. This measurement was conducted by using an acousto-optic modulator (AOM) that alternated the laser beam on and off the device at a frequency of 1 kHz, with a laser power density of 8 mW cm^–2^ and a bias voltage of 10 V. Figure 6d showcases a typical sequence of these on-and-off switching events. It is worth mentioning that the current for the devices stayed stable in repeating measurements with different modulation frequencies. The characteristic rise and fall times were determined by fitting a single-exponential function: *I*_Photo_ = *I*_Photo,0_ + *Ae*^(*t*/τ)^, where *I*_Photo,0_ is the photocurrent at the beginning of rise or fall period, *A* is a pre-exponential constant, *t* is the time, and τ is the rise or fall decay constants. The fittings were done separately to several, single on/off periods, yielding a characteristic exponential rise (or decay) constant that we assign as τ_r_ and τ_d_ for the rise and fall events, respectively. We use this fitting to calculate the rise and fall times, defined as the time consumed to increase from 10% to 90% of the saturation value and vice versa. A typical on–off fitting is shown in Figure 6e, with blue denoting the rise event and red denoting the fall event. Additional fittings done for another device are found in Supporting Information Figure S8. The results collected from four devices indicate a rise time of 52 μs with τ_r_ = 45 μs and a fall time of 120 μs with τ_d_ = 77 μs. The observation of a single exponential decay suggests that the dominant mechanism governing carrier dynamics is their rapid recombination and trapping. Comparing our device performance to other pristine MoS_2_-based devices positions it among the top performing photodetectors. It is outperforming many others as the reported values for *R*_λ_ range from 1 mA/W in MoS_2_ photodiode to 1 kA/W in MoS_2_ phototransistor. Accordingly, the detectivity for the monolayer MoS_2_-based phototransistor has been reported to be in the orders of 10^11^ with rise and fall times in the ranges of 40–110 μs.^69^ This comparison highlights the potential of our MoS_2_ nanofin-nanoribbon hybrid for photodetection, particularly given its simple two-probe configuration.

The fast response exhibited by our devices can be attributed to several factors, including the compact size of the bridging hybrid nanostructure, the application of a high bias voltage, and the minimal separation gap between the source-drain electrodes (3 μm).^35^ According to Rose,^67^ one of the bottlenecks in charge carrier dynamics within a photodetector holds for the presence of shallow trap states near the band edges. Upon exposure to illumination, charge carriers are anticipated to occupy these traps during the rise event, vacating them as the light is switched off, leading to a subsequent reduction in current. We attribute the rapid response to the high crystal purity of our hybrids. Furthermore, it is plausible that the graphoepitaxial in-plane growth method employed here reduces the line-defect concentration within the structure. The guided growth approach allows minimal postgrowth processes, and the MoS_2_ remains chemically pristine and maintains its optimal response.^21^ All of the mentioned factors are reflected in the short rise and fall times, enlightening the advantages of using the guided mixed-dimensional nanostructure in our device.

Finally, we note that the thin nanoribbon significantly influences the photoresponse of the hybrid structure. At the same time, the substantial nanofin size effectively mitigates the impact of surface states throughout the entire structure. This interplay substantially contributes to the rapid charge carrier dynamics and, consequently, the fast response of the optoelectronic device. The MoS_2_ nanofin-nanoribbon hybrid demonstrated a case study in which a mixed-dimensional nanomaterial integrates two components with distinct optical properties, directly impacting the device performance.

## 3 Conclusion

In summary, we report the controlled epitaxial growth of a unique, mixed-dimensional nanostructure of MoS_2_. A nanofin-nanoribbon hybrid combining dimensional features of 1D and 2D MoS_2_ within the same structure is observed by applying a microcavity CVD growth process. The hybrids grow along the three isomorphic m-directions ⟨1̅100⟩of the *C*-plane sapphire substrate, following its 3-fold symmetry. We systematically investigated the hybrid structure’s optical properties, with mapping experiments based on PL and CL measurements showing that the optical response is attributed to excitons generated in the thin nanoribbon. The excitons migrate in the system, resulting in luminescence with different intensities along the sample centered around 680 nm, corresponding to the band-edge emission of bulk MoS_2_. We took advantage of the hybrid dimensions and integrated it as channel material in fin-channel photodetectors. It showed a fast response corresponding to 52–120 μs and a high detectivity of 3.8 × 10^12^ Jones. The device’s performance is attributed to epitaxial growth, without any postgrowth processes, which helps keep the material’s pristine properties. We also integrated the nanofins in finFETs operating at room temperature, manifesting the n-type character of MoS_2_ with an on–off ratio reaching ∼10^3^ and electron mobility of up to 0.4 cm^2^ V^–1^ s^–1^. Sulfur vacancies and high electron concentration explain the electron mobility value relating to excessive scattering events in the material. This can be encountered in future work by improving the contact to lower the resistivity and using high-κ dielectrics. Our work offers a unique mixed-dimensional nanostructure of MoS_2_ with controlled orientations, morphology, and purity, addressing the increasing interest in applications of MoS_2_ in microtechnology, including new device architectures that are not possible in other nanostructures. This method could be expanded to other TMDs, suggesting a variety of epitaxial mixed-dimensional nanostructures with unique solid-state properties.

## 4 Methods

### 4.1 Sample Preparation

MoS_2_ hybrids were prepared using a double source synthesis in a microcavity-based CVD method. We used high-purity MoO_3_ powder (99.999%, Strem Chemicals Inc.) and sulfur chunks (99.998%, Sigma-Aldrich) for the Mo and S sources, respectively. 5 mg of MoO_3_ powder was kept in a quartz boat and placed at the center of a 1-in. tube 2-zone furnace. The *C*-plane and *M*-plane sapphire substrates (Roditi Inc.) were cleaned by sonication in isopropanol (IPA) for 5 min, followed by washing with acetone, IPA, and distilled H_2_O and then blown dry with N_2_; the *M*-plane sapphire was annealed before the synthesis in 1600 °C for 10 h in ambient conditions. The substrate was mounted on top of the same boat facing up. To decrease the deposition rate of MoS_2_ on the substrate, we used a piece of Si/SiO_2_ that was kept on top of the sapphire substrate. Another alumina boat loaded with 500 mg of sulfur chunks was held upstream in a different heating zone, 22 cm away from the MoO_3_ boat. The tube was purged three times with 300 sccm of N_2_ flow at 150 °C and then pumped for 5 min. The temperature then ramped to 750 °C at 15 °C/min with 82–87 sccm of N_2_ flow under ambient pressure. At that time, the S boat was kept at room temperature. When the hot-zone temperature reached 670 °C, the sulfur heating zone was heated to 200–270 °C. The synthesis was carried out for 5 min, and then the furnace was naturally cooled to room temperature. When the MoO_3_ zone was at 560 °C, the sulfur heating zone turned off, and the N_2_ flow rose to 300 sccm.

### 4.2 Structural Analysis

The resulting nanostructures were evaluated with an optical microscope (Olympus BX-51) Nanostructure morphology, size, and shape are profoundly observed using a scanning electron microscope (Sigma 500 SEM, Zeiss) at a working voltage of 3 kV. The crystal structure analysis and growth orientation were done with a focused ion beam (FIB, FEI Helios 600 dual beam microscope, Thermo Fisher Scientific) to cut thin, electron-transparent lamellae in the NW cross-section. This lamella was then observed with a double aberration corrected Temis-Z scanning electron microscope (STEM–Themis-Z). The TEM images were analyzed by extracting reduced FFT patterns from selected areas of the NW cross sections. The *d*-spacing values were compared to literature tables of bulk 2H–MoS_2_ with an error <5%. EDS hyperspectral data were obtained with a Super-X SDD detector through background subtraction and spectrum deconvolution. The height and dimensions of the MoS_2_ crystals were deduced using atomic force microscopy (AFM) (Dimension Edge, Bruker).

XPS was performed using a Kratos Axis Ultra spectrometer, with X-ray intensity of 15 W and 40 eV pass energy, under electron flood gun neutralization.

### 4.3 Optical Characterization

Photoluminescence (PL) and Raman measurements were performed with a micro-Raman/micro-PL spectrometer (Horiba LabRAM HR Evolution) with a 532 nm green laser line The laser was focused on a single hybrid through a reflective objective lens ×100. Band-edge emitted and scattered photons were collected using the same objective and sent to 600 or 1800 lines mm^–1^ grating. PL mapping was done using the same instrument with two-dimensional scanning of a single hybrid. The cathodoluminescence (CL) SEM was done in a Gemini SEM 500 (Zeiss), a high-resolution scanning electron microscope equipped with a two-mode field emission gun. The collected light was directed to a monochromator and a CCD for parallel spectroscopy with a 200–1100 nm spectral range. Simultaneously, an SEM complementary image was collected using the SE2 detector.

### 4.4 Device Fabrication

A photolithography mask was designed to define an electrode pattern with a three-to-five-micron gap to create an array of devices After growth, sapphire substrates with the aligned MoS_2_ hybrid were first marked by standard photolithography. Next, Cr/Au (25/250 nm) metal layers were laid down as electrodes by using electron beam deposition. A photodetector array was obtained after liftoff with acetone. For the fabrication of FETs, we added a dielectric layer and a gate electrode. The dielectric layer was deposited in two stages. At the first stage, a thin layer of aluminum (∼1 nm) was deposited onto the nanofin-nanoribbon channel in an angle evaporation configuration, as explained later in this part. This thin layer was oxidized quickly upon exposure to air, creating the seed for the following Al_2_O_3_ deposition. We then followed the same procedure to fabricate the Au gate electrode (250 nm) after depositing the device with a 50 nm-thick Al_2_O_3_ layer by atomic layer deposition (ALD) (Fiji F200) at 250 °C with tetramethyl aluminum (TMA) and water (H_2_O) as precursors. The gate electrode was deposited in three stages to fit it to the fin-like dimensions of our MoS_2_. First, the sample was positioned on the 45° stage and deposited with a 10/100 nm Cr/Au layer. Then, the sample was rotated at 180° for another 10/100 nm Cr/Au layer deposition cycle. Finally, the sample was positioned on a flat stage for an additional 100 nm of Au deposition.

### 4.5 Optoelectronic and Electrical Characterization

Electrical measurements were done under high vacuum (∼10^–4^ Torr) at room temperature using a Janis ST-500 probe station supplied with a Keithley 4200-SCS measuring system. A 450 nm CW laser illuminated the device with adjustable light intensity. For transient measurements, the beam was periodically shifted on and off the device (modulated) by an acousto-optical modulator (AOM) system with a response time of ∼30 ns.

## Supporting Information Available

MoS_2_ nanofin-nanoribbon hybrid synthesis setup; nucleation centers of MoS_2_ on *C*-plane sapphire; quantitative EDS analysis of MoS2 hybrids on *C*-plane sapphire; XPS measurement of MoS2 hybrids on *C*-plane sapphire; structure and composition of MoS2 nanofins on *A*-plane sapphire; structure and morphology of MoS2 nanofins on annealed *M*-plane sapphire; structure of MoS2 nanofin on annealed *M*-plane sapphire; and exponential fitting to the time-dependent photocurrent in the MoS2 photodetector (PDF)

## Abbreviations

(2D): two-dimensional
(TMDs): transition metal dichalcogenides
(FET): field-effect transistor
(finFET): fin field-effect transistor
(LED): light-emitting diode
(CVD): chemical vapor deposition
(VLS): vapor–liquid–solid
(VS): vapor–solid
(OM): optical microscopy
(FFT): fast Fourier transform
(SEM): scanning electron microscopy
(AFM): atomic force microscopy
(HR-TEM): high-resolution transmission electron microscopy
(XPS): X-ray photoelectron spectroscopy
(EDS): energy dispersive spectroscopy
(PL): photoluminescence
(CL): cathodoluminescence
(PMT): photomultiplier tube
(AOM): acousto-optic modulator
(FIB): focused ion beam
(ALD): atomic layer deposition
(PD): photodetector.
