# Supplementary material for: Epitaxial Mixed-Dimensional MoS2 Nanofin-Nanoribbon Hybrids and Their Integration into Electronic and Optoelectronic Devices
Source: ACS Appl Mater Interfaces. 2025 Apr 30;17(19):28336–49. doi: 10.1021/acsami.5c00308 (PMC12086835; doi:10.1021/acsami.5c00308)
Supplement: Supplementary file 1 — am5c00308_si_001.pdf [file am5c00308_si_001.pdf]

Supporting Information

Epitaxial Mixed-Dimensional MoS_2 Nanofin-Nanoribbon Hybrids and Their Integration into Electronic and Optoelectronic devices

Yarden Danieli[1], Lothar Houben[2], Katya Rechav[2], Olga Brontvein[2], Ifat Kaplan-Ashiri[2], Iddo Pinkas[2], Ayelet Vilan[2] and Ernesto Joselevich[1]*

[1] Department of Molecular Chemistry and Materials Science, Weizmann Institute of Science, Rehovot 7610001, Israel

[2] Department of Chemical Research Support, Weizmann Institute of Science, Rehovot 7610001, Israel

Corresponding author: Ernesto.joselevich@weizmann.ac.il

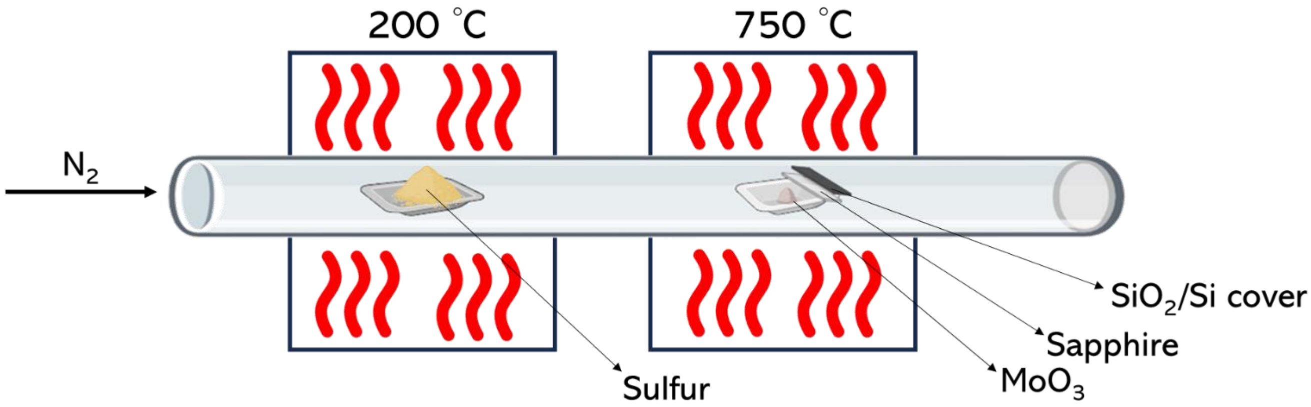

Figure S1. MoS_2 Nanofin-nanoribbon hybrid synthesis setup. The synthesis was conducted using a micro-cavity-based chemical vapor deposition (CVD) method within a quartz tube. To enhance control over substrate deposition, the sapphire substrate was covered with a Si/SiO2 piece. The growth process involved two stages: initially, the temperature of MoO_3 was elevated to approximately 670 °C, followed

by the gradual initiation of sulfur heating to target temperatures of 750 °C and 200 °C respectively. *This Figure was created with BioRender.com*

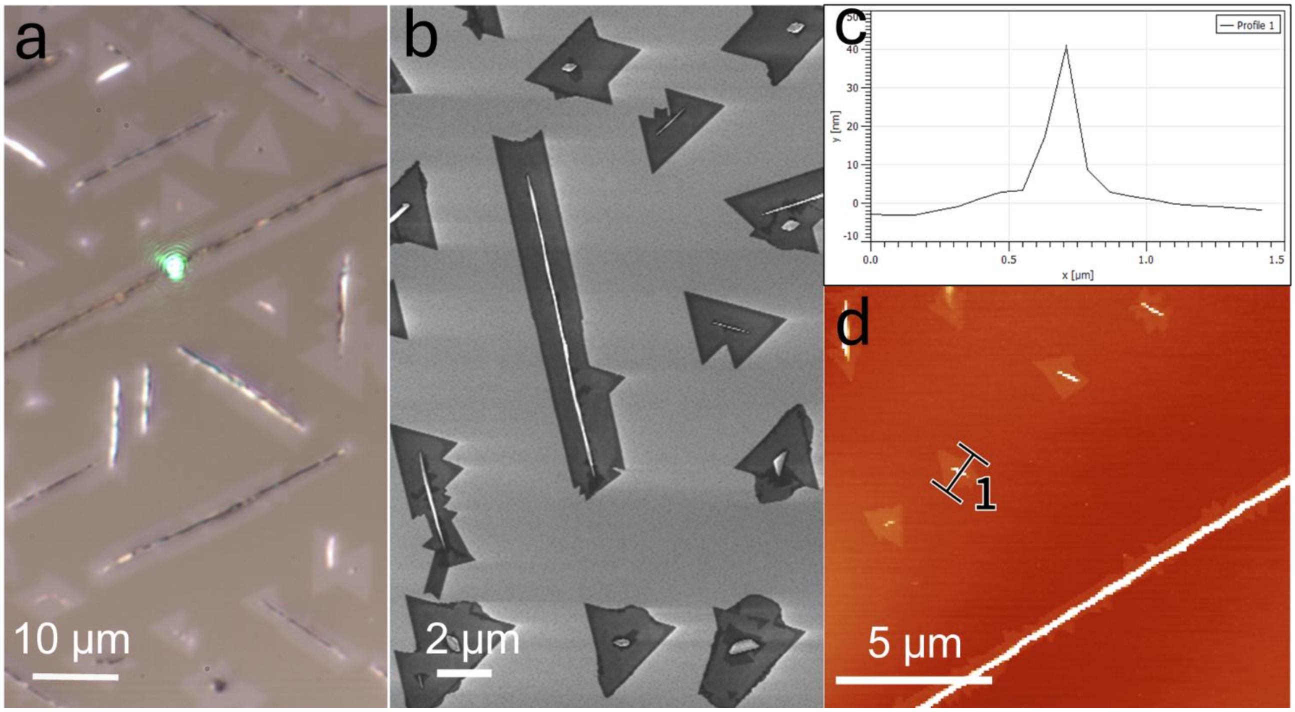

Figure S2. Nucleation centers of MoS_2 on C-plane sapphire. (a) A high resolution and magnification optical microscope image allowing to observe the 1D nanofin in the center of the hybrid, decorated with a 2D nanoribbon around it. This image was taken during a photoluminescence measurement, and the green spot is the laser trace used for the later. (b) An SEM image featuring a 1D nanofin-nanoribbon hybrid alongside a cluster of few-layered triangular flakes of MoS_2. The nucleation center is prominently visible at the center of the flake, and the nanofin serves as a counterpart to these nucleation centers in the 1D hybrids. (c) Height line profile illustrating the dimensions of the flake and the associated nucleation center, as shown in c. (d) An AFM scan of a MoS_2 flake, with a line profile crossing its nucleation center.

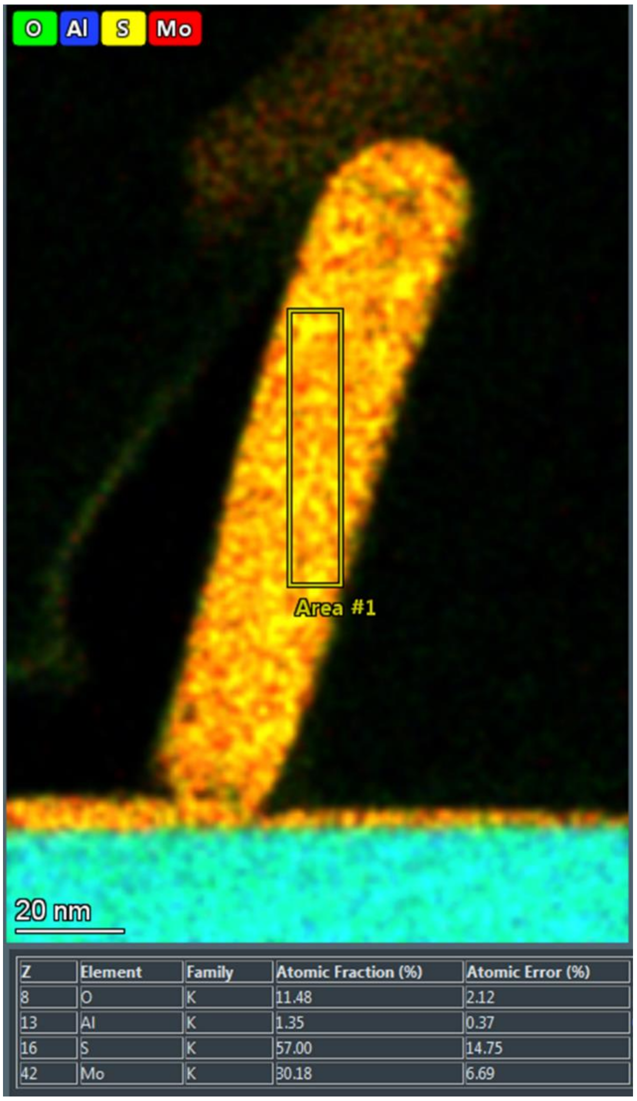

Z	Element	Family	Atomic Fraction (%)	Atomic Error (%)
8	O	K	11.48	2.12
13	Al	K	1.35	0.37
16	S	K	57.00	14.75
42	Mo	K	30.18	6.69

Figure S3. Quantitative EDS analysis of MoS_2 hybrids on C-plane sapphire. a selective area EDS analysis of the MoS_2 nanofin indicating a Mo:S ratio of ~1:2 with agreement to chemical composition of molybdenum disulfide.

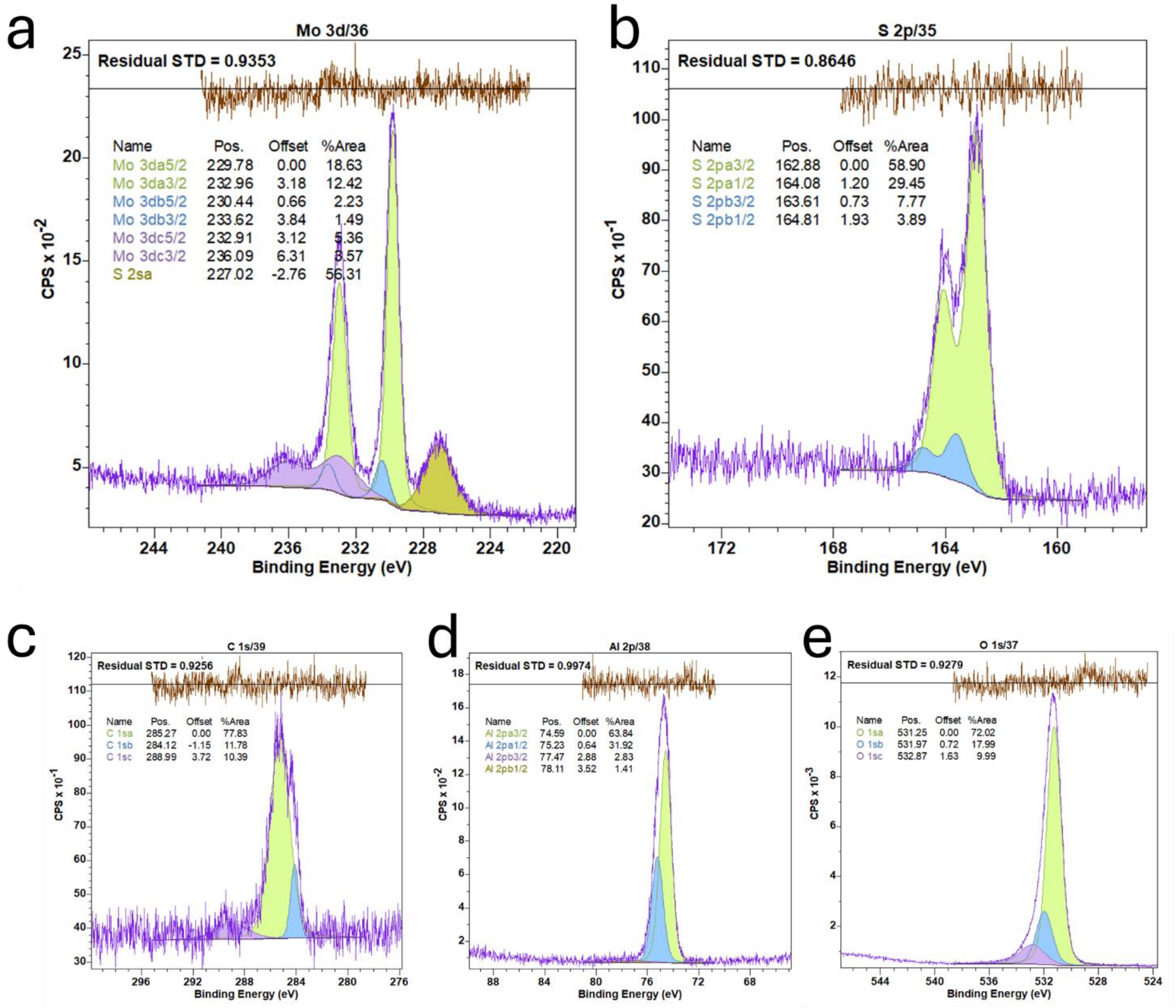

Figure S4. XPS measurement of MoS_2 hybrids on C-plane sapphire. XPS core-level spectra of (a) Mo-3d, (b) S-2p, (c) C-1s, (d) Al-2p and (e) O-1s, collected from MoS_2 nanofin-nanoribbon hybrid grown on C-plane sapphire. Insets are the energy values obtained by the spectra. Binding energy scale is calibrated by setting the C-1s peak center to 285eV. Peak-fitting was done using CasaXPS software, Shirley background and 50% Lorentzian Voight function for peak shape ('LA(50)' line shape of CasaXPS). p- and d-orbitals were fitted by spin-orbit-splitted doublets of standard separations and intensity ratios.

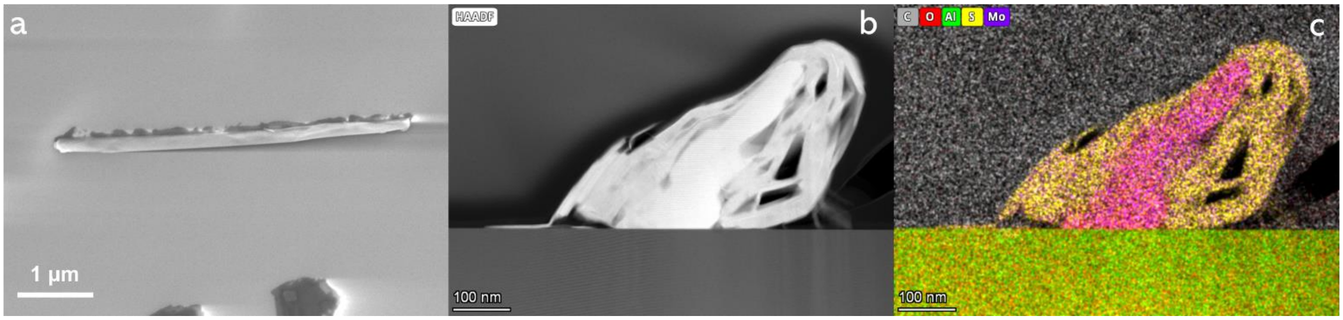

Figure S5. Structure and composition of MoS_2 nanofins on A-plane sapphire. (a) An SEM image of a single MoS_2/MoO_x nanofin grown on A-plane sapphire with the same conditions applied to the growth on C-plane sapphire. (b) A cross-section TEM image of a typical MoS_2/MoO_x nanofin grown on A-plane sapphire, exhibiting a different core and shell phases. (c) An EDS elements map of the nanofin presented in b. The core phase is composed of oxygen (red) and molybdenum (purple) creating a sub-oxide phase, while the shell is layered MoS_2.

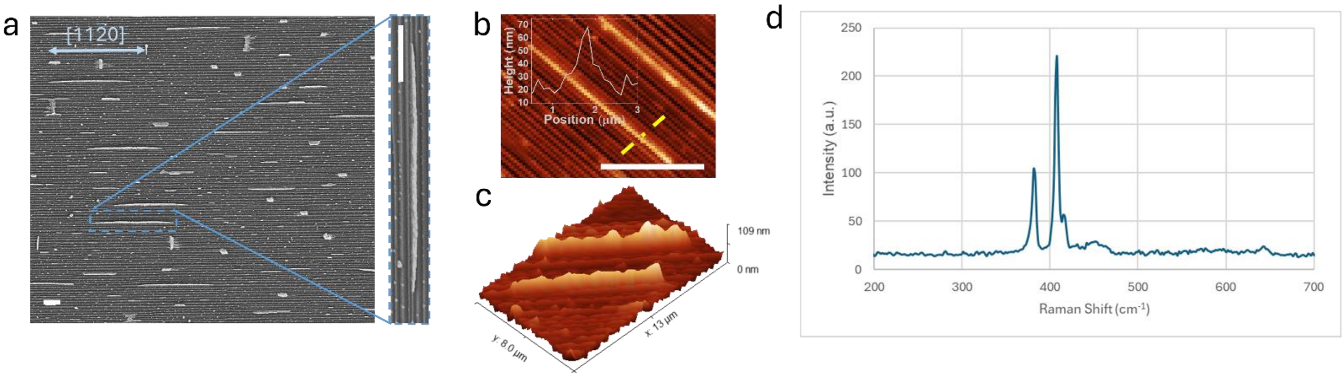

Figure S6. Structure and morphology of MoS_2 nanofins on annealed M-plane sapphire. (a) An scanning electron microscope image showing aligned MoS_2 nanofins on annealed M-plane sapphire, scale is 2 µm; higher magnification image shows a single MoS_2 nanofin with a scale of 2µm. (b) An atomic-force microscope scan of the MoS_2 nanofins on annealed M-plane sapphire. Inset is a height line profile denoted in the figure with a dashed yellow line, scale is 5 µm. (c) A 3D atomic-force microscope image of two MoS_2 nanofins of annealed M-plane sapphire. (d) Raman spectrum of a single MoS_2 nanofin on annealed M-plane sapphire.

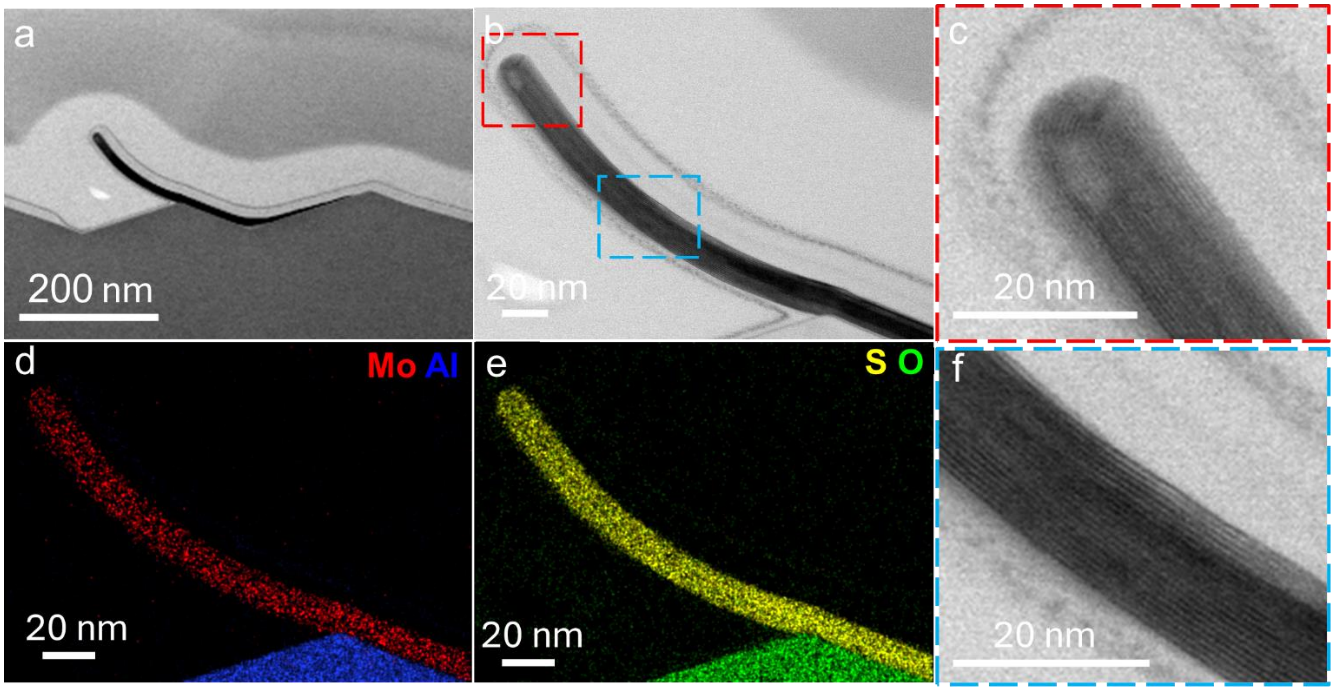

Figure S7. Structure of MoS_2 nanofin on annealed M-plane sapphire. (a) Cross-sectional transmission electron microscope (TEM) image capturing the MoS_2 nanofin grown on annealed M-plane sapphire. (b) A higher magnification TEM image of the nanofin presented in a. (c) and (f) are the corresponding higher magnification TEM images of the edge and nanofin body, in red and blue respectively. (d) Cross-section electron dispersive energy (EDS) map of the hybrid structure, revealing Mo (red) and aluminum (blue) in the sample. (e) Cross-section electron dispersive energy (EDS) map of the hybrid structure, revealing sulfur (yellow) and oxygen (green) in the sample.

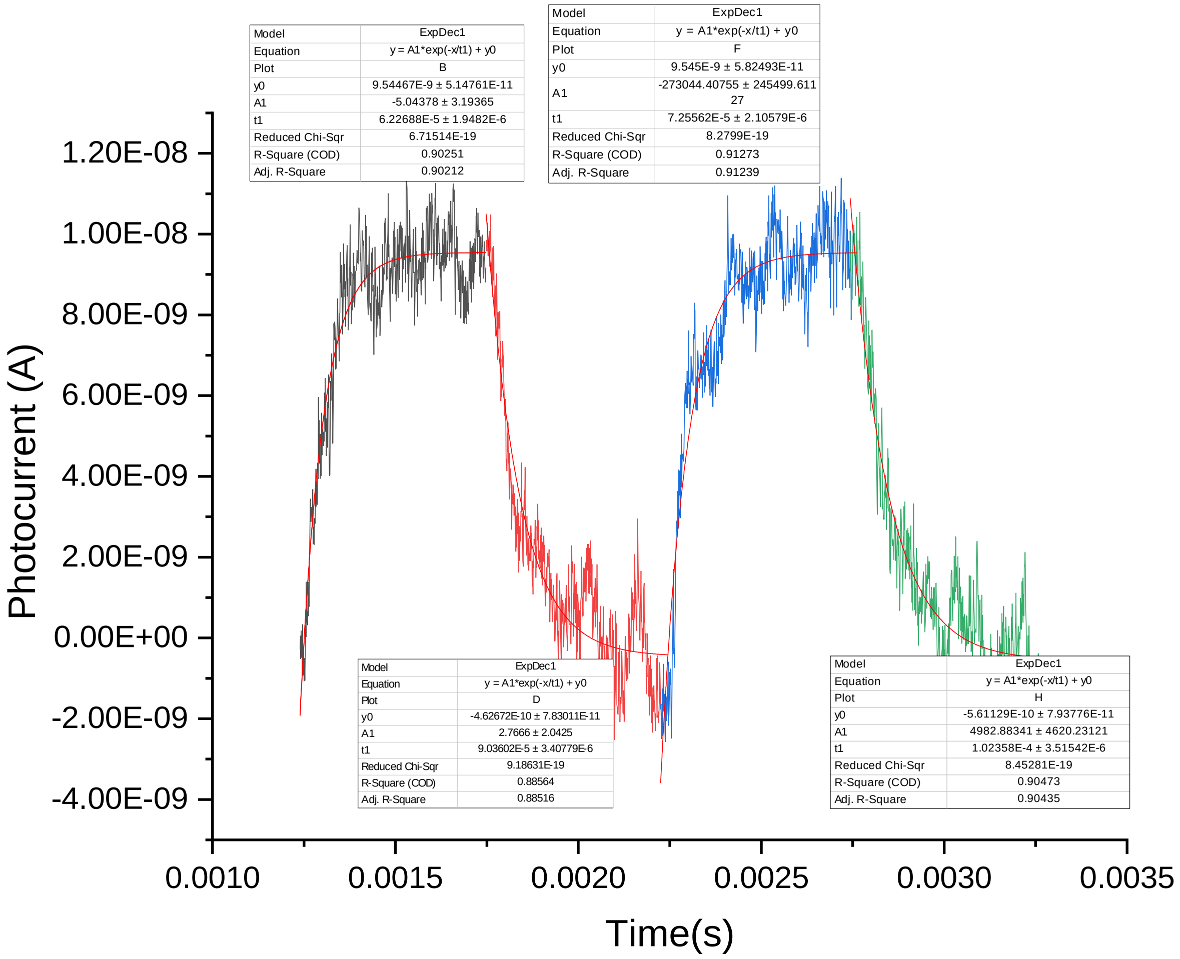

Figure S8. Exponential fitting to the time dependent photocurrent in MoS_2 photodetector. Photocurrent under illumination of a 1 kHz 450 nm laser with a laser power density of 14 mW cm^{-2} 10 V bias. The rise and fall time constants calculated are at the scales of tens of microseconds, as calculated by fitting the rise and fall side with a single exponential function.
